# Lateral Diffusion of a Free Air Jet in Slot-Die Melt Blowing for Microfiber Whipping

**DOI:** 10.3390/polym11050788

**Published:** 2019-05-02

**Authors:** Sheng Xie, Wanli Han, Xufan Xu, Guojun Jiang, Baoqing Shentu

**Affiliations:** 1 College of Material and Textile Engineering, Jiaxing University, Jiaxing 314001, China; jxwlhan@163.com (W.H.); xufanx@126.com (X.X.); 2 State Key Lab of Chemical Engineering, College of Chemical and Biological Engineering, Zhejiang University, Hangzhou 310027, China; shentu@zju.edu.cn; 3 Zhijiang College of Zhejiang University of Technology, Hangzhou 310024, China; jiangguojun1986@126.com

**Keywords:** air diffusion, fiber whipping, melt blowing, hot-wire anemometer, force analysis

## Abstract

In melt blowing, microfibrous nonwoven material is manufactured by using high-speed air to attenuate polymer melt. The melt-blown air jet determines the process of polymer attenuation and fiber formation. In this work, the importance of lateral velocity on the fiber was first theoretical verified. The lateral diffused characteristic of the air flow field in slot-die melt blowing was researched by measuring the velocity direction using a dual-wire probe hot-wire anemometer. Meanwhile, the fiber path was captured by high-speed photography. Results showed that there existed a critical boundary of the lateral diffusion, however, air jets in the *x–z* plane are a completely diffused field. This work indicates that the lateral velocity in the *y–z* plane is one of the crucial factors for initiating fiber whipping and fiber distribution.

## 1. Introduction

Melt blowing is an industrial approach for manufacturing nonwoven materials. Melt-blown nonwoven materials have found a variety of advanced applications in areas of filtration, life science, medicine, and industry [1,2], the fiber diameter is commonly in the range from about 1 μm to several micrometers.

**Figure 1:**
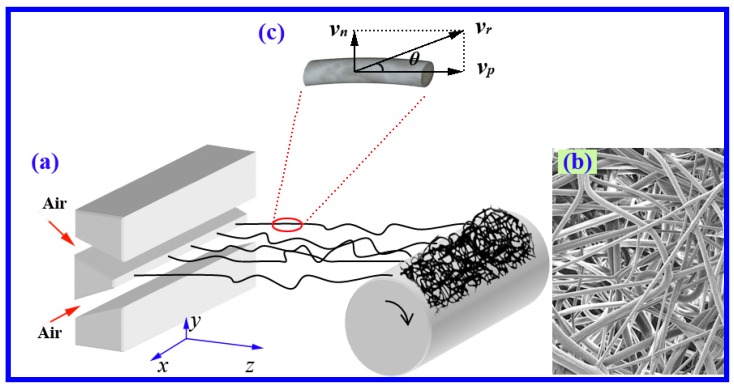
(**a**) Schematic of commercial slot-die melt-blown process. (**b**) The microfibrous nonwovens produced by melt blowing. (**c**) Schematic of a fiber fragment near the die and the air velocity applied on it.

During the melt blowing process, the raw material of solid particles is melted by the effects of heating and the screw’s shear stress, and subsequently extruded out through the orifice, the microfibers are formed by attenuating the melt with high-velocity hot air (see Figure 1). Therefore, the air flow field plays a decisive role on fiber attenuation (formation) during melt blowing. Shambaugh et al. [3,4,5,6] have relatively early measured the air velocity and temperature by Pitot tube and thermocouples (or infrared thermometer), respectively, in the condition of relatively low air velocity. Later, more-advanced measuring equipment like laser Doppler velocimeters and hot-wire anemometers with one-dimensional probes were applied for measuring the melt-blown air flow fields [7,8,9,10,11], the maximum air velocity they measured increased to be about 150 m/s. By means of the high-frequency signal acquisition principle of the hot-wire anemometer, Xie [11] measured the fluctuation of turbulence in melt blowing and discussed the relationship between turbulence and fiber motion. It noted that, air velocity is a vector and has the characteristics of directivity, however, these previous experimental measurements provided the velocity along the spinning line, the directional information of velocity was unavailable, especially for positions not along the spinning line, generally, these measured velocities were considered to have a direction parallel to the spinning line. Therefore, the air flow field was researched with a lack of information along the lateral direction.

In melt blowing, the air is pushed out from air channels and ejected into the surrounding static atmospheric, therefore, the melt-blown air flow needs to be a free jet and have the feature of diffusion, that is, the cross-section of the air flow is ever increasing along the spinning line direction. The diffusion of air flow could be demonstrated by a lot of Computational Fluid Dynamics (CFD) simulations [9,12] and indirectly reflected by the splayed-out envelope of the fiber path, which is attributed to fiber vibration or whipping in the process of melt blowing [13,14,15]. A bundle of fibers that appeared to be splaying rather than a single fiber was firstly captured by Shambaugh [13]. This observation was similar to that from the naked eye. Similar splayed-out envelopes of fiber paths were also experimentally discovered by Bresee et al. [14] and Xie [15]. The increased envelope of the fiber path is assumed by the lateral air velocity component (direction normal to the spinning line), however, until now, the measurement of the lateral velocity component has not been reported and most of the air flow fields were measured along the spinning-line direction.

In addition, the theoretical research on fiber motion is important, for it can deeply reveal the stretching mechanism of fiber attenuation in the spinning process, not only for melt blowing but also for other process such as electrospinning [16]. The theory of the bending instability of thin liquid jets in air was developed and described by Yarin [17]. Rao [18] and Marla [19] built two- and three-dimensional theoretical models for fiber attenuation in melt blowing, respectively. Compared to the former one-dimensional model [20], the normal drag force was applied onto the fiber. Bates, Kumar, and Macosko from the University of Minnesota [21,22,23,24,25,26,27] have done some theoretical and experimental work on fiber formation, special applications, and innovative designing of melt blowing. Kumar [23] firstly point out that, in melt blowing, the whipping-like motion of the fiber was due to turbulent air, this guidance promoted our previous work on the turbulence of melt blowing [11,15]. Yarin et al. [28,29,30,31,32] have numerical modeled the process of fiber motion and predicted the structure and properties of melt-blown nonwoven lay-down. Their works are assumed to have a crucial effect on quality control in melt blowing. Sun [33] built a Euler–Lagrange model for the mechanics simulation of fiber motion during the melt-blown process, and this model was improved to make the fiber path more realistic by Xie et al. [34] These theoretical models focused on mechanical mechanisms, rather than citing detailed air information of the whole air flow field. Therefore, the discovery of more consummate flow field information will contribute to perfecting these theoretical researches.

It is well known that the melt-blown air’s resultant velocity can be decomposed into vertical and lateral components, i.e., their directions are parallel and normal to the spinning line, respectively. The present work focused on discovering the lateral component of the air flow in slot-die melt blowing. Firstly, the significance or necessity of measuring the lateral velocity was theoretical verified. The theoretical result showed that the lateral component had high efficiency on fiber dragging, and should not be ignored. Then, the air velocity direction was measured by a dual-wire hot-wire anemometer, and the lateral air velocity component was obtained by processing the measured velocity direction, meanwhile, the fiber path during melt blowing was captured by high-speed photography. Finally, the characteristics of lateral diffusion were shown, and the potential effect of lateral diffusion on fiber whipping was discussed.

## 2. Theoretical Verification the Importance of Lateral Velocity

Figure 1a shows the schematic process of melt blowing, in which microfibers are manufactured by attenuating the polymeric melt with high-velocity hot air. As a whole, the fibers will fly along the spinning line and downward to the collector. Figure 1b shows the microfiber nonwovens manufactured by melt blowing. In this work, the spinning line is the indicated *z*-axis, and the lateral direction is normal to the spinning line, i.e., along the *x* or *y* direction.

Intuitively, the lateral air component is very small, this may be the reason why the lateral component was neglected by these previous works, which have been mentioned above. As will be described below, the importance or necessity of lateral component measurement was firstly verified by theoretical force analysis.

This theoretical verification was carried out by analyzing the air force on the fiber. As shown in Figure 1c, a random tiny fragment of fiber was chosen for modeling. During the process of melt blowing, it was assumed that the fiber fragment has a two-dimensional resultant velocity, *v_f_*, which can decomposed into parallel and normal components with respect to the axis-direction of the fiber, i.e., *v_fP_* and *v_fN_*. Similarly, the air applied on the fiber fragment has a resultant velocity, *v_r_*, by decomposing *v_r_* into parallel and normal components with respect to axis-direction of the fiber, *v_P_* and *v_N_* are obtained. The angle between *v_r_* and the axis of the fiber is *θ*. The relative velocity of the air and the fiber, *v_rel_*, can decompose into

(1a) $$v_{rel,P}=v_{r}\cos(\theta )-v_{fP}\;(0\;\leq \;\theta \;\leq \;180^{{}^{\circ}}),$$

(1b) $$v_{rel,N}=v_{r}\sin(\theta )-v_{fN}\;(0\;\leq \;\theta \;\leq \;180^{{}^{\circ}}),$$

where *v_rel,P_* and *v_rel,N_* are the component velocities of the relative velocity, which are parallel and normal to the axis of the fiber.

Components velocities of *v_rel,P_* and *v_rel,N_*, respectively, create accordingly a “parallel drag force”, *F_P_*, and a “normal drag force”, *F_N_*, on the fiber [18]. The appropriate definitions for the two drag forces are

(2a) $$F_{P}=\frac{1}{2}C_{P}\rho_{a}{\left(v_{rel,P}\right)}^{2}(\pi d_{f}l_{f}),$$

(2b) $$F_{N}=\frac{1}{2}C_{N}\rho_{a}{\left(v_{rel,N}\right)}^{2}(d_{f}l_{f}),$$

where *C_P_* and *C_N_* are the skin coefficient and the drag coefficient, respectively; *ρ_a_* is the density of air; and *d_f_* and *l_f_* represent the diameter and length of the fiber fragment, respectively. Additionally, there are also the following relationships [35,36,37].

(3a) CP=0.78(ReDP)−0.61,

(3b) CN=6.96(ReDN)−0.440(df/d0)0.404,

(4a) ReDP=ρavrel,Pdfμa,

(4b) ReDN=ρavrel,Ndfμa,

where *μ_a_* is the kinematic viscose of air; *d*_0_ is 7.8 × 10^−5^ m [37].

Substituting Equations (3a) and (4a) into Equation (2a), and substituting Equations (3b) and (4b) into Equation (2b), *F_P_* and *F_N_* are

(5a) $$F_{P}=0.39\pi \frac{{\rho_{a}}^{0.39}{\left(v_{r}\cos(\theta )-v_{fP}\right)}^{1.39}{d_{f}}^{0.39}l_{f}}{{\left(\mu_{a}\right)}^{-0.61}}$$

(5b) $$F_{N}=159\frac{{\rho_{a}}^{0.56}{\left(v_{r}\sin(\theta )-v_{fN}\right)}^{1.56}{d_{f}}^{0.964}l_{f}}{{\mu_{a}}^{-0.440}}.$$

Because the fiber velocity is much smaller than the air velocity [15], here, *v_f_* is simplified to be 0 m/s, i.e., *v_fP_* and *v_fN_* are all zero. Substituting *ρ_a_* =1.293 kg/m^3^, *μ_a_* = 0.297 × 10^−4^ Pa·s, and assuming *d_f_* = 0.42 × 10^−3^ m, *l_f_* = 10^−3^ m, and *v_r_* = 100 m/s in Equation (5), the relationships between *F_P_*, *F_N_*, and *θ* are

(6a) $$F_{P}=6.81\times 10^{-5}\cos^{1.39}(\theta )\;(0\leq \theta \leq 180^{{}^{\circ}})$$

(6b) $$F_{N}=1.40\times 10^{-3}\sin^{1.56}(\theta )\;(0\leq \theta \leq 180^{{}^{\circ}}).$$

**Figure 2:**
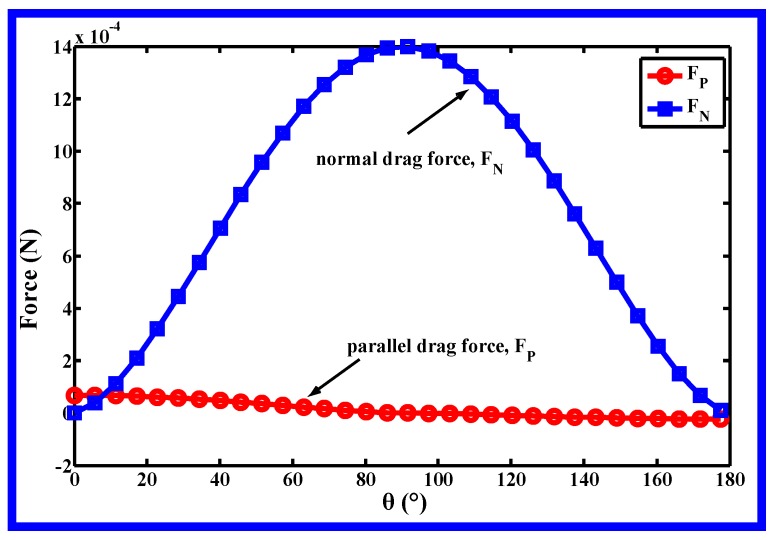
*Fp*, *F_N_* with respect to *θ* under the same assumption.

Figure 2 shows *F_P_*, *F_N_* with respect to *θ* under the same assumption. The *F_N_* is much higher than the *F_P_* at the same *θ*, especially when *θ* is around 90°. The force analysis of the fiber illustrates that even if the lateral velocities, *v_x_* and *v_y_*, are not as large as the spinning-line-direction velocity component, *v_z_* [11], lateral velocities have higher efficiency on dragging fibers. Additionally, the force analysis shows the measurement of lateral diffusion is meaningful.

## 3. Experiments

### 3.1. Melt-Blown Setups

**Figure 3:**
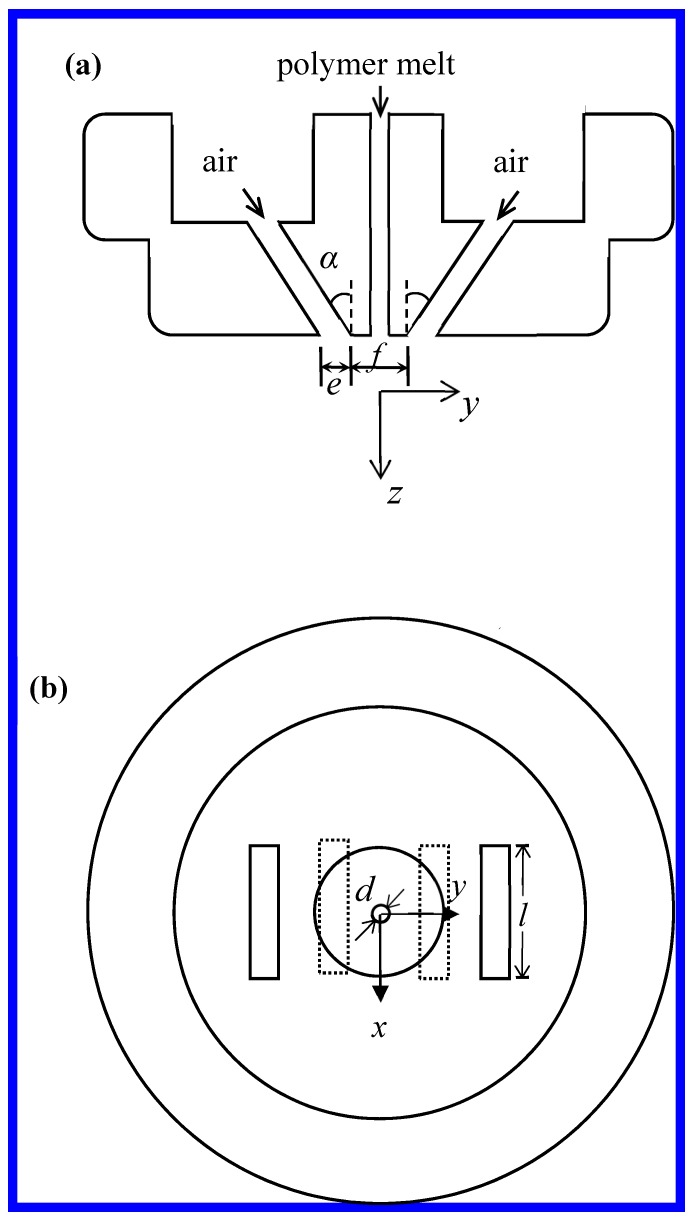
(**a**) Section view (**b**) top view of the slot die used in this work.

The measurement of air velocity direction in this study was performed on the single-orifice slot-die melt-blown device (if the multifibers, shown in Figure 1a, are changed to be single fiber, then the single-orifice slot-die melt-blown process is shown). The structure of the slot die used in this work is shown in Figure 3. Slot die is referred to as a blunt-edge with a nose-piece width (*f*) of 1.28 mm, a slot angle (*α*) of 30°, and a slot width (*e*) of 0.65 mm, the orifice diameter for the polymer melt (*d*) was 0.42 mm. Other details of this slot die can be found in the manuscript of Xie [15], this kind of die was also described by other researchers [3,10]. The coordinate system used is also shown in Figure 3, all coordinates are relative to the die face. Its origin is at the center of the die face, the *x* direction is along the major axis of the nose piece and slots, whereas the *y* direction is transverse to the major axis of the nose piece and slots. The *z* direction is directed vertically downward.

### 3.2. Air Velocity Direction Measurements

**Figure 4:**
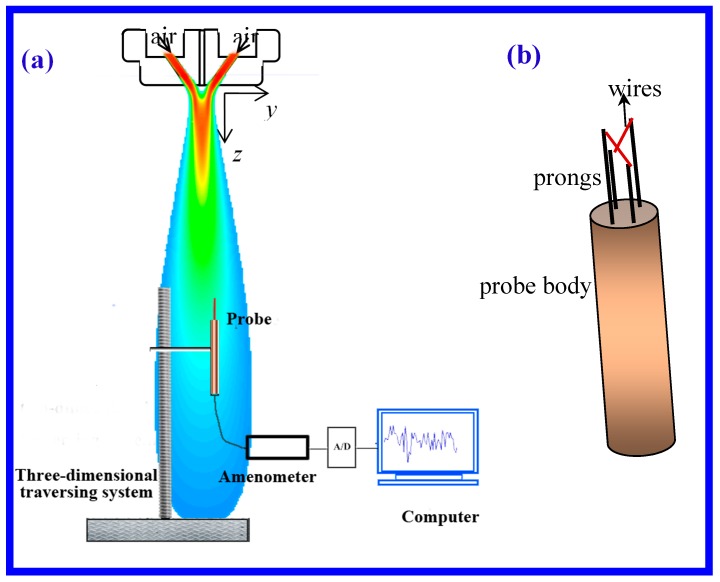
(**a**) Schematic of the constant temperature anemometer (CTA) device and melt-blown die during air velocity direction measurement. The shown air flow field is obtained by Computational Fluid Dynamics (CFD). (**b**) The structures of the dual-wire probe.

As shown in Figure 4a, the velocity direction below the die was online measured with a hot-wire anemometer (Dantec StreamLine CTA90C10, Dantec Dynamics, Skovlunde, Denmark) in the absence of the polymer stream. A gauge air pressure of 1.0 atm was supplied for measurement. This anemometer is often called CTA or constant temperature anemometer with reference to the operating principle. The CTA works on the basis of convective heat transfer from a hot wire to the surrounding fluid, the heat transfer being primarily related to the fluid velocity, namely, the melt-blowing air takes away the heat of the wire, the temperature of the wire is kept constant by applying voltage on it. The relationship between air velocity and the applied voltage is

(7) $$\frac{E}{R_{w}}=[\frac{\left(R_{w}-R_{f})(A+Bv^{1/2}\right)}{\alpha_{0}R_{f}R_{w}}],$$

where *E* is the heating voltage applied on the wire; *R_w_* and *R_f_* are the resistance of the wire at temperature of wire and air, respectively; A and B are constant numbers; *v* is the air velocity; and α_0_ is the temperature coefficient of resistance.

The CTA signal was acquired via an A/D converter board and saved as a data series in a computer. Series of air velocities were obtained by converting the data series of voltages via Equation (7). Actually, the so-called air velocity refers to the mean of the data series of velocities. As a core component of the CTA, probes are available in one-, two-, and three-dimensional versions as single-, dual-, and triple-wire probes referring to the number of wires. Since the wires respond to both magnitude and direction of the velocity vector, information about both can be obtained, only when two or more wires are placed under different angles to the flow vector. A dual-wire probe was attempted for the velocity-direction measurement in this work, Figure 4b illustrates the structure of the dual-wire probe used in this measurement, the dual-wire probe consisted of two systems of single-wire probes, each probe had a wire with a 5 μm diameter and a 1.6 mm length spot-welded to needle-shaped prongs, made of stainless steel. The two planes which contain the two wires and their corresponding prongs were parallel to each other, the projections on the parallel planes of the two wires were vertical. Note that the arrangement of the two wires was very important during measurement, for example, the plane which contains the two wires and its corresponding prongs is in the *x–z* plane, with this arrangement, the angle between the projection of the air velocity in the *x–z* plane and the *z*-axis is measured. In the present work, the projection of the air velocity in the *x–z* plane was called velocity in *x–z* plane for short.

During the measurements of air velocity direction, the probe was positioned with a traversing system, which permitted upward or downward motion along the *z*-axis in 2 mm increments. In consideration of probe protection, the minimum distance below the die was set to *z* = 6 mm. The measurement was carried out on 384 discrete positions, these discrete positions were along eight series lines, they were

Positions along line 1: [*x* = 0, *y* = 2, *z* = 6, 8, 10, … 100 mm]

Positions along line 2: [*x* = 0, *y* = 5, *z* = 6, 8, 10, … 100 mm]

Positions along line 3: [*x* = 0, *y* = 10, *z* = 6, 8, 10, … 100 mm]

Positions along line 4: [*x* = 0, *y* = 20, *z* = 6, 8, 10, … 100 mm]

Positions along line 5: [*x* = 2, *y* = 0, *z* = 6, 8, 10, … 100 mm]

Positions along line 6: [*x* = 5, *y* = 0, *z* = 6, 8, 10, … 100 mm]

Positions along line 7: [*x* = 10, *y* = 0, *z* = 6, 8, 10, … 100 mm]

Positions along line 8: [*x* = 20, *y* = 0, *z* = 6, 8, 10, … 100 mm]

**Figure 5:**
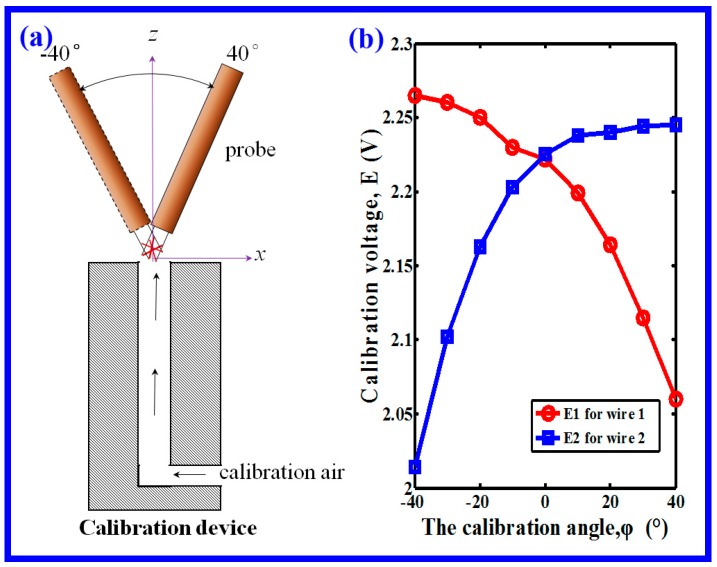
(**a**) Schematic of devices for the directional calibration of the CTA. Directional calibration was performed by exposing the probe (wires) to a known air velocity from different angles. (**b**) Calibration angle and its two corresponding voltages for the dual wires.

Calibration is an indispensable process before all formal measurements, similarly, before the air velocity direction measurement, the directional calibration was accomplished on a calibration device at ambient temperature. Calibration establishes a relationship between the CTA output and the air direction. As shown in Figure 5a, it was performed by exposing the probe to a known air velocity from different angles, two responding voltages (*E*_1_ and *E*_2_) could be obtained for the dual wires. By the principle of Equation (7), *E*_1_ and *E*_2_ were different, because of the angle of arrangement of the dual wires. Figure 5b shows the calibration angle and its two responding voltages for the dual wires, which is called the directional-calibration curve, the calibration angle, *φ*, was set from −40° to 40° with an interval of 10°. Here, the calibration angle and the ratio of *E*_1_ and *E*_2_ had a relationship of

(8) $$\varphi =\frac{1.0066-E_{1}/E_{2}}{0.0023}{\;R}^{2}=0.9954.$$

Later, during the formal measurement, the air velocity direction could be calculated from the ratio of output of *E*_1_ and *E*_2_ according to Equation (8). In this measurement, the discovery of lateral diffusion was carried out by measuring the velocity direction in two typical vertical planes, i.e., *y–z* and *x–z* plane.

### 3.3. Fiber Path Measurement

Polypropylene (SK, Seoul, Korea) with melt flow rate of 650 g/10 min was used in the fiber spinning process. The base conditions were a polymer flow rate of 7.1 g/min, a polymer temperature of 260 °C, and a gauge air pressure of 1.0 atm with room temperature.

The melt-blown fiber motion was online captured by a high-speed camera (Redlake Inc., San Diego, CA, USA), which has the capability of recording images at a frame rate up to 100,000 partial frames/s. The camera was equipped with a Nikon 24–85 mm, *f* 2.8 zoom lens. Two 2000 W lamps were added as a light source to make the fiber more clear. Fiber paths in a region of 62.5 mm × 130 mm in the *y–z* plane and the *x–z* plane were captured, respectively, at the frame rate of 1500 frames/s. Similar measurements of the fiber path can be found in the previous work of Xie [15] or Shambaugh [38].

## 4. Results and Discussion

### 4.1. Air Velocity Direction

**Figure 6:**
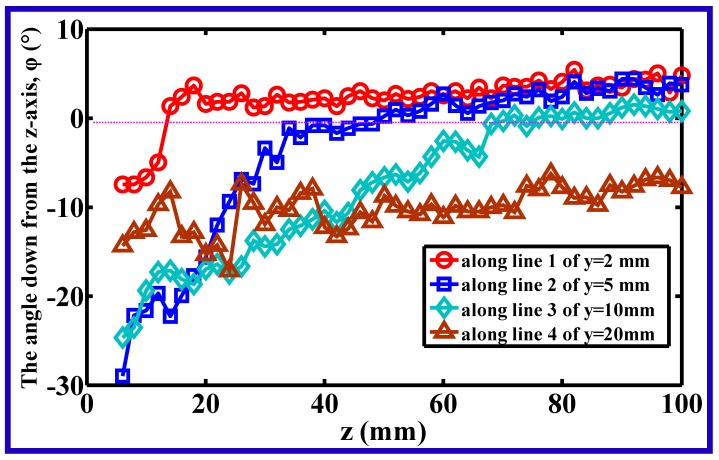
The directional angle between the *z*-axis and the resultant velocity in the *y–z* plane at different position along lines 1–4. Lines 1–4 were defined as described in Section 3.2.

Figure 6 shows the directional angle between the *z*-axis and the resultant velocity in the *y–z* plane (here, resultant velocity in the *y–z* plane means the projection of the air resultant velocity on *y–z* plane), at different positions along lines 1–4 (lines 1–4 correspond to the lines of *y* = 2, 5, 10, and 20 mm in the *y–z* plane). The resultant velocity in the *y–z* plane consisted of the component velocities of *v_y_* and *v_z_*, where *v_y_* was definitely the lateral component velocity.

Along line 1 of *y* = 2 mm, the angle down from the *z*-axis, *φ*, was about −8° at *z* = 6 mm, and increased to be a positive value at a critical position of *z* = 14 mm, the angle had a small increase in the distance beyond *z* = 14 mm, but all of them were less than 5°. Similarly, the angles along lines 2 and 3 of *y* = 5 and 10 mm, had minimum (negative) values at *z* = 6 mm, and increased with increasing distance from the die, at critical positions of *z* = 50 and 72 mm, the values of their angle changed to be positive. For the angle along line 4 of *y* = 20 mm, although the angle had a general increasing trend with increasing distance from the die, there existed a large angle fluctuation near the die, especially in the region of *z* < 40 mm, revealing that the velocity in the *y–z* plane became unstable in the lateral region far away from the spinning line, i.e., the *z*-axis. The reason for the instability was deduced to be caused by the collision of the air jet and the surrounding atmosphere. In Figure 6, the positive value represents that the direction of the resultant velocity deviates from the *z*-axis, i.e, *v_y_* has a positive direction along the *y*-axis. Similarly, the negative value meant the *v_y_* had opposite direction with the *y*-axis. According to this illustration, an interesting phenomenon can be discovered, namely that there exists a veer of lateral velocity in the slot-die melt-blown air flow field, which means that there exists a critical boundary of the lateral diffusion of air flow in the *y–z* plane during slot-die melt blowing.

**Figure 7:**
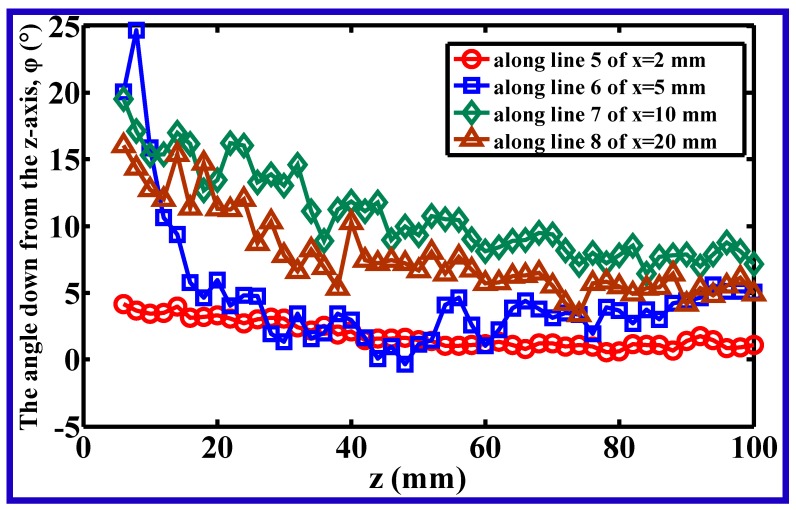
The angle down from the *z*-axis of the resultant air velocity in the *x–z* plane at different positions along lines 5–8.

Figure 7 shows the directional angle between the *z*-axis and the resultant velocity in the *x–z* plane, at different positions along lines 5–8 (lines 5–8 correspond to the positions of *x* = 2, 5, 10, and 20 mm in the *x–z* plane). The resultant velocity in the *x–z* plane consisted of component velocities of *v_x_* and *v_z_*, *v_x_* is also the so-called lateral velocity in this work. Unlike the profiles of angles shown in Figure 6, all angles in Figure 7 are positive in value, indicating that the air jets in the *x–z* plane are a completely diffused field. Moreover, the angles commonly decrease with increasing distance from the die, implying that the lateral diffused effect decreases with increasing distance from the die.

**Figure 8:**
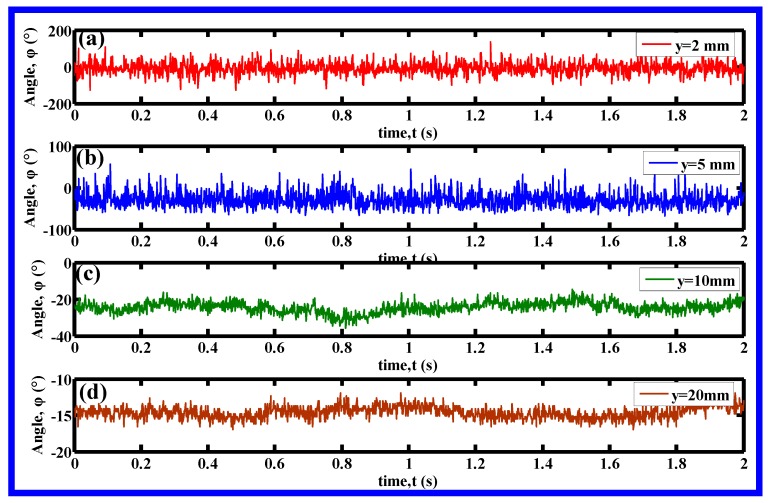
The fluctuant signals of the angle between the *z*-axis and the resultant velocity in the *y–z* plane at the (*y*, *z*) positions of (**a**) (2 mm, 6 mm), (**b**) (5 mm, 6 mm), (**c**) (10 mm, 6 mm), (**d**) (20 mm, 6 mm).

Figure 8 shows the fluctuant signals of the angle between the resultant velocity in the *y–z* plane and the *z*-axis, at four lateral discrete positions, i.e., (*y*, *z*) = (2, 6), (5, 6), (10, 6), and (20 mm, 6 mm), respectively. A time segment of 2 s was chosen for the demonstration of the angle’s fluctuation. As shown in Figure 8, these fluctuant signals are irregular and different at different positions. These fluctuation signals were expected to contain lots of potential information, however, the investigation of the potential information of signals is not the topic of this work, therefore, the fluctuation signals at the other 380 positions (the total positions in this work is 384) are not shown in this part. Even so, it is certain that the polymeric fiber undergoes pulsating air force during melt blowing, which might be one reason for initiating fiber instability during melt blowing.

### 4.2. Lateral Velocity Components: v_y_ and v_x_

**Figure 9:**
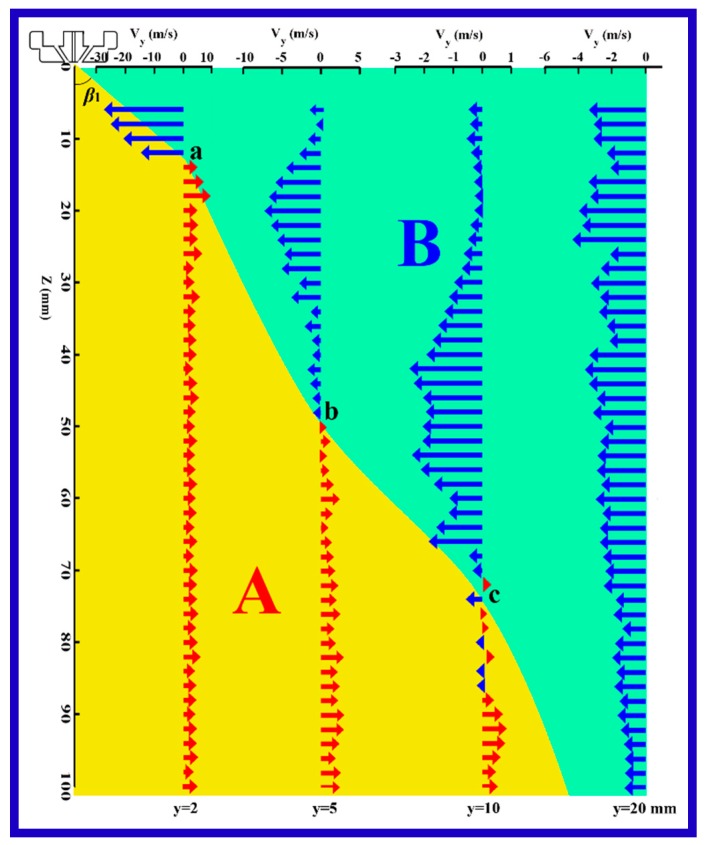
The distribution of lateral velocity, *v_y_*, along lines 1–4 of *y* = 2, 5, 10, and 20 mm, in the corresponding *y–z* plane. A represents the lateral diffusion region, B represents the lateral back-diffusion region.

Figure 9 shows the distribution of the lateral velocity, *v_y_*, along lines 1–4 of *y* = 2, 5, 10, and 20 mm, in the corresponding *y–z* plane. Here, the lateral velocity component was calculated via multiplying resultant velocity by the angles, which are shown in Figure 6 and Figure 7. It should be pointed out that Figure 9 has a double abscissa, the upper and the lower abscissa are *v_y_* and *y* positions, respectively. For the *v_y_* along line 1 of *y* = 2 mm, the *v_y_* had a magnitude of 28 mm/s and had back-diffusion direction within the positions above the point “a” signed in this figure, however, the *v_y_* had a lateral diffused direction at positions below the critical point “a”. Here, ”a” is deemed to be the so-called boundary of veer, which has been mentioned above. At positions along lines 2 and 3 of *y* = 5 and 10 mm, *v_y_* has similar profiles to *v_y_* along line 1, moreover, the maximum value of *v_y_* decreased laterally far away from the *z*-axis, and the boundary points of veer are signed “b” and “c”, respectively. However, for the *v_y_* along line 4 of *y* = 20 mm, all *v_y_* has back-diffusion direction, even though the maximum *v_y_* is as small as about 4.5 m/s. In a word, as shown in Figure 9, air flow has the characteristic of lateral diffusion in region A (yellow region), and lateral back-diffusion in the region B (green region). To further represent the lateral diffused characteristic of air flow in the *y–z* plane, a diffused envelope angle, *β*, is built, which is defined by Equation (9) below. Furthermore, *β* is calculated to be about 17.1°.

(9) $$\beta =2\left(\frac{\beta_{1}+\beta_{2}+\beta_{3}}{3}\right),$$

where *β*_1_ is the angle between the *z*-axis and the line segment of origin and “a”; similarly, *β*_2_ is the angle between the *z*-direction and the line segment of “a” and “b”, and *β*_3_ is the angle between the *z*-direction and the line segment of “b” and “c”. Here, the *y–z* coordinates of “a”, “b”, and “c” are (2, 14), (5, 50), and (10 mm, 72 mm).

**Figure 10:**
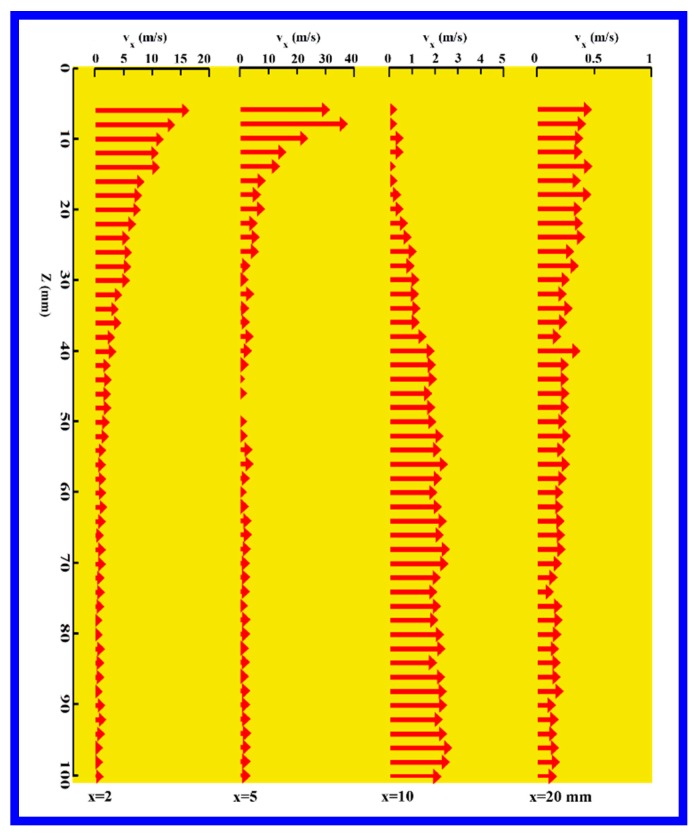
The distribution of lateral velocity, *v_x_*, along lines 5–8 of *x* = 2, 5, 10, and 20 mm, in the corresponding *x–z* plane.

Figure 10 shows the distribution of lateral velocity, *v_x_*, along lines 5–8 of *x* = 2, 5, 10, and 20 mm in the *x–z* plane. Unlike the distribution of lateral velocity, *v_y_*, shown in Figure 9, all *v_x_* have direction along positive the *x* direction, showing that there is no critical boundary of lateral *v_x_* in the *x–z* plane. At the position nearest to the orifice of the die face, the maximum value of *v_x_* is about 15 m/s, which is approximately half of *v_y_* = 28 m/s, indicating that the polymer melt, which has just been squeezed out from the orifice, undergoes higher lateral velocity along *y* direction than along *x* direction. This phenomenon deserves to be an import reason for the anisotropic distribution of fiber path in the *x–y* plane, which will be further discussed below.

### 4.3. Fiber Path in Melt Blowing

**Figure 11:**
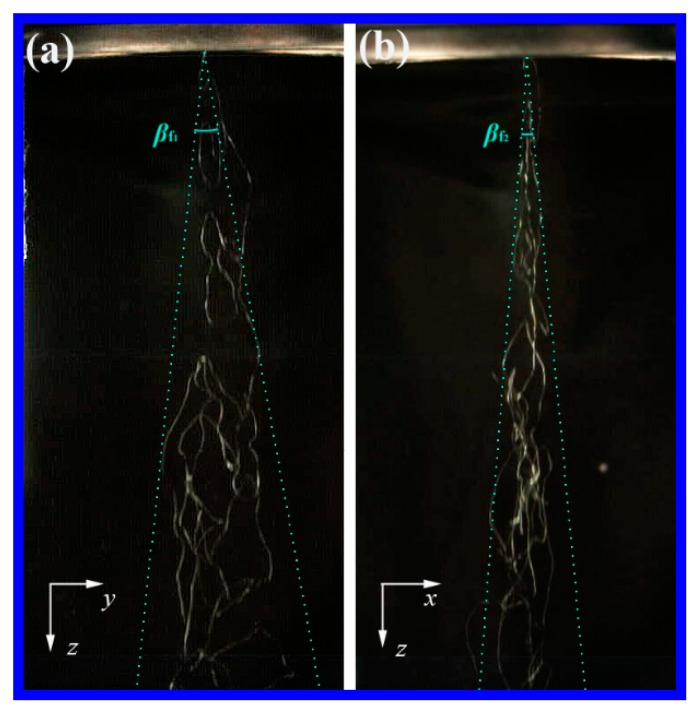
The fiber paths below the melt-blown slot die. (**a**) The fiber path in the *y–z* plane. (**b**) The fiber path in the *x–z* plane. The corresponding size of each image is 62.5 mm × 130 mm.

Figure 11 shows the fiber paths below the melt-blown slot die. It is obviously that the cross-section of the fiber path in the *x–y* plane is not round (but more likely to be elliptical), the amplitude of the fiber path in the *y–z* plane is larger than that in the *x–z* plane. The envelope angle of the fiber path in the *y–z* plane, *β*_f1_, is about 16.3°, while, the envelope angle in the *x–z* plane, *β*_f2_, is 9.1°. It is worth noting that *β*_f1_ = 16.3° is fairly consistent with the air diffused envelope angle, *β* = 17.1° in the *y–z* plane, indicating that the critical boundary of the air lateral diffusion might control the lateral fiber distribution during melt blowing, or at least they have a close relationship.

During melt blowing, fiber whipping occurs as soon as the polymer melts out from the orifice, according to Yarin’s theory [17], when the flow velocity incident on a jet, exceeds a critical velocity, a small disturbance of the jet will grow and develop into a bending instability. Figure 9 and Figure 10 provide an extra clue for initiating fiber whipping, fiber is driven directly by the lateral velocity all the time without critical velocity mentioned by Yarin [17]. In consideration of the high drag efficiency of lateral velocity on the fiber (shown in Figure 2), there are reasons to believe that the small lateral velocity still has a crucial effect on initiating instability or whipping of the polymeric fiber during melt blowing.

## 5. Conclusions

In this work, the necessity of measuring the lateral velocity in melt blowing was first theoretical verified. Then, the velocity direction of the air flow in slot-die melt blowing was measured using a dual-probe hot-wire anemometer, and the fiber path was captured by high-speed photography. The results of the air velocity direction show that there exists a critical boundary of air lateral diffusion in the *y–z* plane. However, the air flow in the *x–z* plane is a completely diffused field. In addition, the fluctuating signal of the velocity direction is also shown, which might be a reason for initiating fiber instability. Finally, the relationship between the air lateral diffusion and the fiber whipping path was compared and discussed.

This work provides an extra clue for initiating fiber whipping, the lateral velocity plays crucial effect on initiating instability or whipping of the polymeric fiber, even though it is much smaller than the vertical velocity. This work indicates that, the air lateral diffusion deserves to be further discovering for detailed melt-blown research.
